# Non-Conventional Induction Heat Treatment: Effect of Design and Electrical Parameters on Apple Juice Safety and Quality

**DOI:** 10.3390/foods11233937

**Published:** 2022-12-06

**Authors:** Shilin Wu, Xueming Xu, Na Yang, Yamei Jin, Zhengyu Jin, Zhengjun Xie

**Affiliations:** 1 College of Biological and Food Engineering, Anhui Polytechnic University, Wuhu 241000, China; 2 School of Food Science and Technology, Jiangnan University, 1800 Lihu Road, Wuxi 214122, China; 3 State Key Laboratory of Biobased Material and Green Papermaking, Qilu University of Technology, Shandong Academic of Sciences, Jinan 250301, China

**Keywords:** non-conventional induction heating, structure design, thermal processing, liquid food

## Abstract

The proposed non-conventional induction heating, which combines an MSCP and VDC structure, was proved to have excellent thermal effect. Different from other electric field sterilization, this electrotechnology operates with no electrodes, and it is a continuous-flow process with short-duration (about 20 s). In current study, the parameters related to temperature rise were investigated, including applied voltage, frequency, the diameter of the secondary coil and heating tube, as well as their length, etc. It was demonstrated that a smaller diameter of the heating tube, parallel connection sample coils, and higher frequency were beneficial for the inactivation of microorganisms. At 500 Hz, the optimal condition is 800 V, *d*_1_ = 2 mm, and *L*_1_ = 10 cm. Notably, the system could inactivate all microorganisms and maintained the physicochemical properties of apple juice at 40 kHz. It suggests that this structural design has the potential for industrial applications and the proposed induction heating can realize the rapid sterilization of liquid food without applying electrodes.

## 1. Introduction

The conventional method used to sterilize liquid food is thermal pasteurization, which has been developed for decades [1], it is also considered to be an effective and economical sterilization method in the food industry [2]. Although there are many studies about non-thermal processing technologies, thermal treatment is dominant with foodborne pathogens inactivation. In addition, the literature demonstrated that non-thermal processing technology combined with moderate temperature had a good performance compared with the non-thermal treatment alone [3,4]. Among existing electrotechnologies such as ohmic heating (OH) [5] and pulsed electric fields (PEF) [6], except for enabling food safety, they are used to maintain food quality. Meanwhile, those systems are equipped with metal electrodes. PEF treatment has a significant non-thermal effect [7], as well as synergy effect [8]. Recently, Wu et al. [9] presented innovative induction heating that could sterilize liquid food by IEF, without inserting electrodes. This concept and process of the treatment were thus illustrated.

Currently, thermal technologies applied in food processing mainly include conduction heating [10], electrical heating [11,12], and microwave heating [13]. Heat conduction technology treats food through steam or hot water [14,15,16]. As for electrical heating, food is heated when electric current passes through it [17]. The principle of microwave heating is dominated by dielectric properties of water molecules and the food component at a specific frequency [18]. Different from these technologies, the proposed induction heating is based on electromagnetic induction, and the winding coil of liquid food is then subjected to an alternating magnetic field [19]. Then, a liquid sample passes through a sample coil, an induced electric field (IEF) is produced inside the sample, then it is heated simultaneously. In addition, the treatment is a continuous-flow process that meets the direction of industrialization. In a previous study [9], the process principle was discussed. However, the effect of structural parameters, were not pointed out. Thus, it is necessary to explore the thermal effect of IEF caused by the design and electrical parameters.

In this study, the structure of MSCP (Multiple Secondary coils Connected in Parallel) and VDC (Variable Diameter Connection) was proposed. In addition, we investigated the parameters influencing the thermal effect caused by the IEF via recording the temperature profile in apple juice. Furthermore, the physicochemical properties and microorganisms of apple juice were also determined in order to evaluate the EIH technology on the sterilization application for the production.

## 2. Methods and Materials

### 2.1. IEF Generator

**Figure 1:**
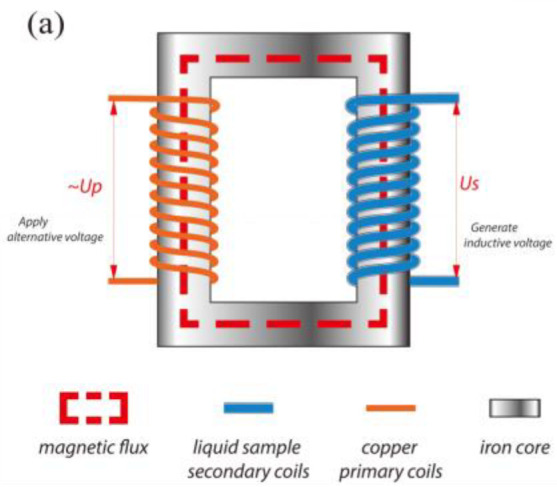

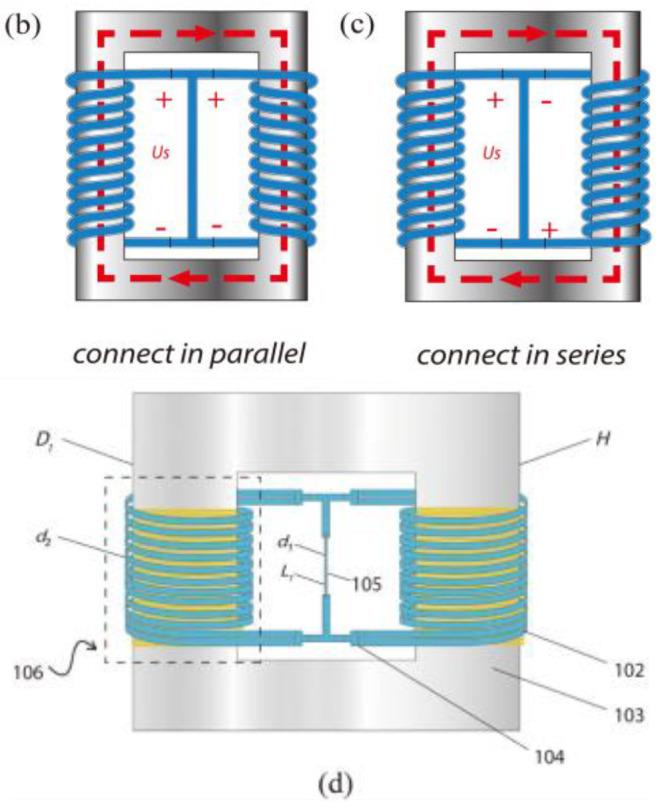
(**a**) Principle of IEF generator; (**b**) Parallel connection; (**c**) Series connection; (**d**) IEF generator with MSCP and VDC structure.

The principle of the IEF generator is presented in Figure 1a. There are two different connections of secondary coils (or sample coils) containing a parallel and series connection (Figure 1b,c). The parallel connection is applied since it can induce a large potential difference between the link tube (or the heating tube). In the current study, the proposed IEF generator structure was illustrated at Figure 1d. Furthermore, 102 is the secondary coil (made of insulation material, poly tetra fluoroethylene) where it was full of samples, 103 is the iron core, 104 is the plastic three-way valve, 105 is the link tube, 106 is one of the winding units (it contains two pairs of secondary coils connected in parallel as well as a primary coil); *D*_1_ and *H* is the diameter and height of the magnetic circuit, respectively; *d*_1_ is the diameter of link tube and *d*_2_ is the diameter of secondary coil; *L*_1_ is the length of the link tube. In the current study, the system is equipped with two winding units (2 × *N_S_* = 2 × 9 or 2 × 10, *N_S_* is number of primary coils), *d*_2_ = 8 mm, *D*_1_ = 32 cm, *H* = 80 cm, *L*_1_ =10 cm, *N*_P_ (number of primary coils) = 48 was applied.

### 2.2. Structures of MSCP and VDC

The structures of VDC and MSCP are illustrated in Figure 1d, where *d*_2_ is larger than *d*_1_, and the *N_S_* is unlimited. This structure resulted in the increase of potential difference between the link tube 105. Several secondary coils were (positive to positive and negative to negative) connected in parallel to ensure that output voltage improved with the increase of the *N*_S_. According to the circuit principle, the induced current *I*_Total_ (also the current in the link tube) is the sum of the induced currents in each sample coil.

The MSCP and VDC structures were investigated. The VDC led to the increase of induced voltage as the diameter difference between the sample coil and link tube enlarged, which amplified the thermal and non-thermal effect. Besides, the structure of MSCP had a significant performance on temperature rise, which clarified the enlargement of the current in the parallel circuit of the liquid sample, rather than the series circuit [19]. Therefore, the combined MSCP and VDC structure could heat liquid food rapidly.

### 2.3. Operation Principle

**Figure 2:**
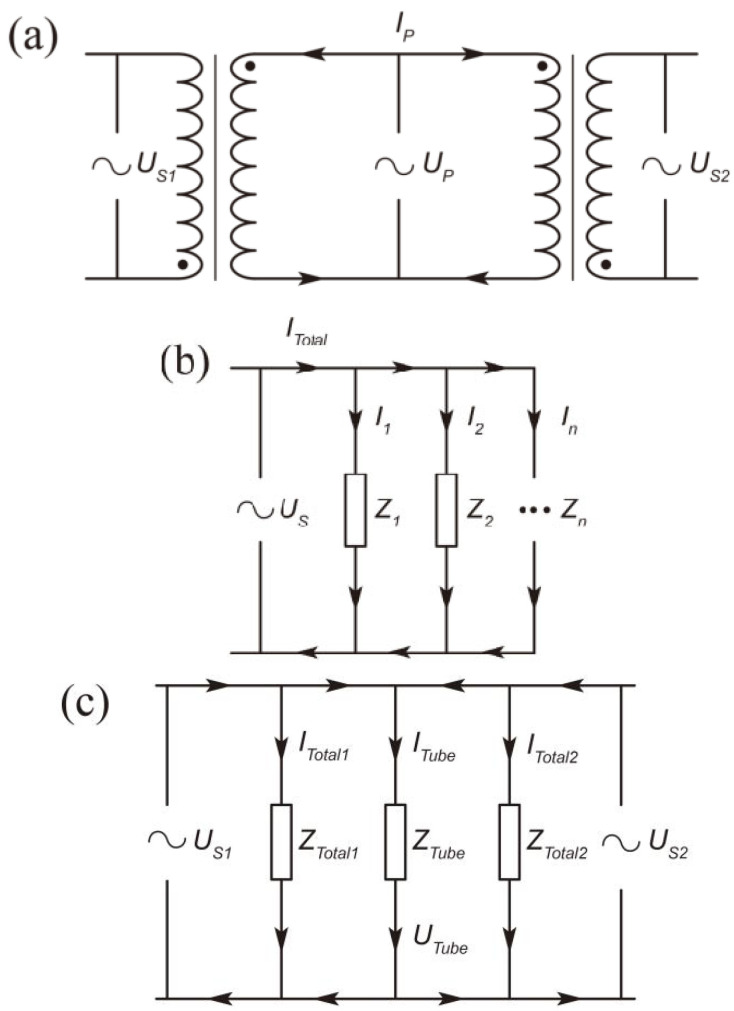
Equivalent circuit of primary and secondary coils (**a**), 106 unit (**b**), and IEF system (**c**).

According to the structure of MSCP, the system operation principle is shown in Figure 2a. The *U*_S1_ and *U*_S2_ are the induced voltages in the secondary coils S1 and S2, respectively, between the link tube, they vary as the applied voltage in the primary coils (*U*_P_) change. According to the theory of transformer [20], the relationship between *U*_S1_, *U*_S2_ and *U*_P_ can be expressed as Equations (1) and (2):

(1) $$\frac{U_{S1}}{U_{P}}=\frac{N_{S1}}{N_{P}}\cdot \omega$$

(2) $$\frac{U_{S2}}{U_{P}}=\frac{N_{S2}}{N_{P}}\cdot \omega$$

where *ω* is a constant (*ω* < 1), mainly depending on the physicochemical properties of liquid food filling in the secondary coils.

The equal circuit of 106 is shown in Figure 2b and the total resistance (*Z*_Total_) of this unit is calculated as Equation (3):

(3) $$Z_{\mathrm{Total}}=\frac{Z_{1}Z_{2}\cdot \cdot Z_{n}}{Z_{1}+Z_{2}\cdot \cdot +Z_{n}}$$

where *n* is the number of secondary coils. According to the equation, as the *Z*_Total_ of MSCP decreased, the *ω* is increased owing to *U*_P_ maintained at a constant level, which results in the increase of *U*_S1_ and *U*_S2_. Eventually, the induced current (*I*_Total1_ and *I*_Total2_) in each secondary coil’s unit (106) is increased.

Theoretically, the voltage (*U*_Tube_) between the link tube is equal to *U_S_*_1_ and *U_S_*_2_ based on its structure (Figure 2c). According to the parallel circuit, the current in the link tube *I*_Tube_ could be calculated via Equation (4):

(4) $$I_{\mathrm{Tube}}=I_{\mathrm{Total}1}+I_{\mathrm{Total}2}$$

thus, *I*_Tube_ is increased under the structure of the MSCP.

In the VDC structure, the *d*_1_ is related to current density *J*. By Equation (5), it can be seen that smaller *d*_1_ causes higher current density.

(5) $$J=\frac{I_{\mathrm{Tube}}}{S}=\frac{4I_{\mathrm{Tube}}}{\pi \cdot d_{1}^{2}}$$

The thermal effect of IEF can be described as *W*, which is correlated with the current density (*J*). The relationship between them is expressed by Equation (6):

(6) $$J\propto W=k\cdot \frac{\Delta T}{\Delta t}=k\cdot \frac{T_{i}-T_{0}}{t_{i}-t_{0}}$$

which *k* is a constant and *i* is the duration. Therefore, it suggests that the thermal effect can be improved with the reduction of *d*_1_.

In summary, the structure of MSCP reduces total resistance of secondary coils and the VDC structure increases the current density, and then improves the thermal effect of IEF.

### 2.4. Sample Preparation

Apple juice (AJ) was purchased from a local supermarket (WeiChuan Food Co., Ltd., Hangzhou, China). The AJ was mixed with 20% (*m*/*v*) potassium chloride (AR Grade, Sinopharm Reagent Co., Ltd., Shanghai, China) obtaining a specific conductivity, as this level is a major factor during electrical treatment. In order to ensure the initial temperature (25 °C), before the treatment, all samples were kept in a refrigerator at 4 °C for 30 min.

The samples were treated with different voltages (200 V, 500 V and 800 V) and frequencies (300 Hz, 400 Hz and 500 Hz). Instead, the other group was treated at 40 kHz and 1800 V. All samples passed through the link tube for inner heating for about 20 s.

### 2.5. Temperature Rise

The temperature was measured with an infrared thermal camera (C3, FLIR Systems Inc., Wilsonville, OR, USA), thus the temperature of the link tube (105) was recorded. Since the temperature rose significantly in the first 3 min and then remained at a terminal level. For the evaluation of the heating process, it defined the parameters *T*_mean_ and *V*_mean_ by Equations (7) and (8):

(7) $$T_{\mathrm{mean}}=\frac{T_{4}+T_{5}+\ldots +T_{10}}{7}$$

(8) $$V_{\mathrm{mean}}=\frac{T_{3}-T_{0}}{3}$$

where *T*_mean_ is the mean value of the temperature in the last seven minutes; *V*_mean_ is average temperature rise in the first three minutes. *T*_0_, *T*_1_ … *T*_10_ are temperature at each minute.

### 2.6. Conductivity, pH, Brix, Impedance and Resistance

The physicochemical properties of the samples were analyzed. The conductivity was detected using a conductivity meter (FE28, Mettle-Toledo Instruments Co., Ltd., Shanghai, China), pH was measured via a pH meter (FE38, Mettle-Toledo instruments Co., Ltd., Shanghai, China) and the Brix value was recorded by a portable refractometer (WZS 20, Shanghai Yidian Physical Optical Instrument Co., Ltd., Shanghai, China). The impedance and resistance were measured using an impedance analyzer (6500B, Wayne Kerr Electronics, Bognor Regis, West Sussex, UK) at *H*_gap_ = 2 mm.

### 2.7. Microorganisms Counting

The samples were placed in a darkroom for 6 h at room temperature until microorganism concentration reached 10^4^–10^5^ CFU·mL^−1^. Before placing them into an IEF instrument, the sample was stirred by a magnetic stirrer to make it homogeneous. After the treatment, the sample was collected and stored at 4 °C before counting. The microorganism content in each sample was determined using the plate count method as previously reported by Char et al. [21] with a slight modification. A solution of 8.5% (*w*/*v*) saline was prepared for serial dilutions, and the diluted samples were uniformly plated on plate count agar (PCA, Qingdao Hope Bio-Technology Co., Ltd., Qingdao, China). Then these plates were incubated in a thermostatic incubator (HYL-C, Qiangle Experimental Equipment Co., Ltd., Taicang, China) at 37 °C for 2 days. Eventually, the result was calculated using the following formula (Equation (9)):

(9) $$N_{\mathrm{IEF}}=\frac{C_{0}-C_{i}}{C_{0}}\times 100\%$$

where *N*_IEF_ is the sterilization efficiency, *C*_0_ is the initial concentration of bacteria in the sample, whereas *C*_i_ is that of the treated sample.

### 2.8. Statistical Analysis

All experiments were performed in triplicates. The data were analyzed by one-way analysis of variance (ANOVA) tests using the SPSS 17.0 software (IBM Corporation, Armonk, NY, USA). The results are expressed as the mean value ± standard deviation.

## 3. Results and Discussions

### 3.1. Different Connections and the Diameter of the Link Tube

**Figure 3:**
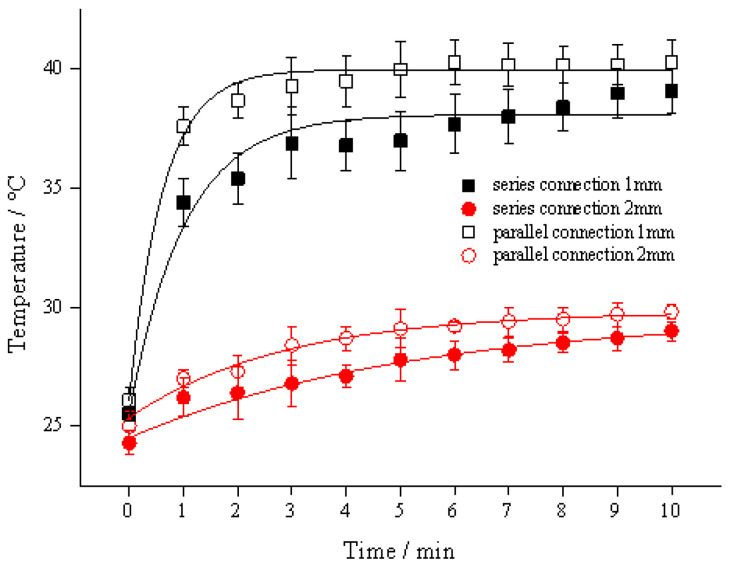
Temperature profile of apple juice fulfilled in sample coils with different connections at 800 V, 400 Hz, *d*_2_ = 8 mm. Note: two connections were displayed with *N*_S_ = 2 × 19.

The results are presented in Figure 3, it was demonstrated that the heating effect caused by the parallel connection of the sample coil was better than that of the connection in series. The *T*_3_ of *d*_1_ = 1 mm and *d*_1_ = 2 mm in the parallel structure (39.3 °C and 28.4 °C) are 2.4 °C and 1.6 °C higher than those in the series structure (36.9 °C and 26.8 °C), respectively. This was due to the reduction of impedance level in the system with parallel structure on the sample coil, namely *Z*_Total_ in Equation (3). In the literature [22], the reactor units were connected in series and utilized for acid hydrolysis of starch. Their results showed that the IEF could significantly improve the hydrolysis efficiency compared with the control group (by stirring or immersion) at an output voltage of 15 V. In addition, the proposed coil structure with a parallel connection had better hydrolysis performance than that of the series structure. The reason why the sample in the parallel structure had higher temperature rise, was that the parallel circuit enlarged induced current in the link tube. In addition, the *V*_Tube_ on the parallel structure is 800 V when the *V*_S1_ and *V*_S2_ both are 800 V, whereas the *V*_Tube_ on the series structure might be 400 V as the potential is averaged within the circuit (Figure 2c).

At the same time, *d*_1_ played an important role in this induction heating. For the parallel structure, the group with *d*_1_ = 1 mm is 38.4% (10.9 °C) higher than that of 2 mm. As shown in Figure 3, the IEF produced in liquid food caused the temperature rise with short duration, and its terminal temperature increased slightly. The trend is similar to OH [11]. Both OH and IEF treatment are inner heating techniques, and the terminal temperature is determined by sample properties and output power [12]. In addition, the flow rate of the sample in the heating chamber also influences the terminal temperature, which relates to its retention time.

These results show that the parallel connection of the sample coils reduced the *Z_coil_* which was conducive to the heating.

The data in Table 1 revealed that the influence of the *d*_1_ on IEF treatment was significant in both the AJ and AJ + 20% KCl samples, and the temperature increased with the decrease of the *d*_1_ level. For the AJ group, at *d*_1_ =1 mm, 2 mm, 3 mm and 5 mm, the *T*_2_ increased by 11 °C, 3.1 °C, 1.2 °C and 1.1 °C compared with the *T*_0_, respectively. In the AJ + 20% KCl group, at *d*_1_ = 2 mm, 3 mm and 5 mm, the *T*_2_ increased by 33 °C, 28.1 °C and 18 °C, respectively. Notably, the temperature of the sample reached 78.5 °C from room temperature within one minute (*d*_1_ = 1 mm), and bubbles formed inside the link tube due to high temperature. It was indicated that bright sparks were produced as the shaking of bubbles. Similarly, air ionization and spark production in the heating chamber have been reported during the OH process [23]. These bubbles become deformed around the chamber [24], which lead to the formation of a temperature gradient and then some areas did not reach the boiling point [23].

According to Equations (5) and (10), the decreased *d_1_* causing high temperature could partly be explained by the increase of current density. In addition, the reduction of cross-sectional area of the treatment chamber leads to the increase of its resistance.

(10) $$R=\rho \frac{l}{S}$$

where *R* is the resistance, *ρ* is the resistivity of the liquid sample, *l* is the length of the link tube, and *S* is the cross-sectional area of the link tube.

The Joule heating effect is positively correlated with current density [19]. Consequently, the temperature increased with the decrease of *d*_1_. This phenomenon is more significant in the AJ + 20%KCl group. Furthermore, terminal temperature of the juice was above 80 °C as *d*_1_ = 1 mm, which indicated that the conductivity was also an impact factor for this innovative induction heating.

### 3.2. Primary Voltage and Conductivity

The results in Table 2 show that the primary voltage had a positive impact on the thermal effect. In the AJ group, the *T*_10_ at 800 V was of 2.6 °C higher than that of 200 V, whereas the temperature was 38.3 °C in the AJ + 20% KCl group. With the increase of applied voltage, the thermal effect was more significant. This phenomenon was consistent with PEF treatment and OH results [17,25]. The reason was that the *U*_Tube_ and *I*_Tube_ would increase simultaneously with the improvement of the *U*_P_ at a certain Z_Tube_, enhancing the current density and accelerating the heating rate as well as terminal temperature. Actually, the thermal effect occurs in electrotechnologies that are determined by the field strength [26,27]. On the other hand, low conductivity of liquid food cannot be heated under the IEF, thus the MSCP and VDC structure are necessary.

At 800 V, the *T*_2_ of the AJ + 20% KCl group (*κ*_s_ = 224.5 mS/cm) rose to 58.5 °C, that was 33 °C higher than the *T*_0_, while *T*_10_ increased to 70 °C. With the increase of the conductivity, the terminal temperature increased concurrently. The *T*_10_ of the AJ group increased by 0.5 °C, while that of the AJ + 20% KCl group rose to 11.5 °C compared with the *T*_2_ at 800 V. In addition, the temperature rise with increasing conductivity has also been reported in other studies on electric field processing [28,29]. As the conductivity increased, the overall impedance decreased which caused the increase of induced current. Although the temperature of the sample remained within a limited variation range after 10 min, it showed a slight growth trend. The increased conductivity of the sample was observed at high temperature [30].

### 3.3. Number of Parallel Secondary Coils (N_S_)

**Figure 4:**
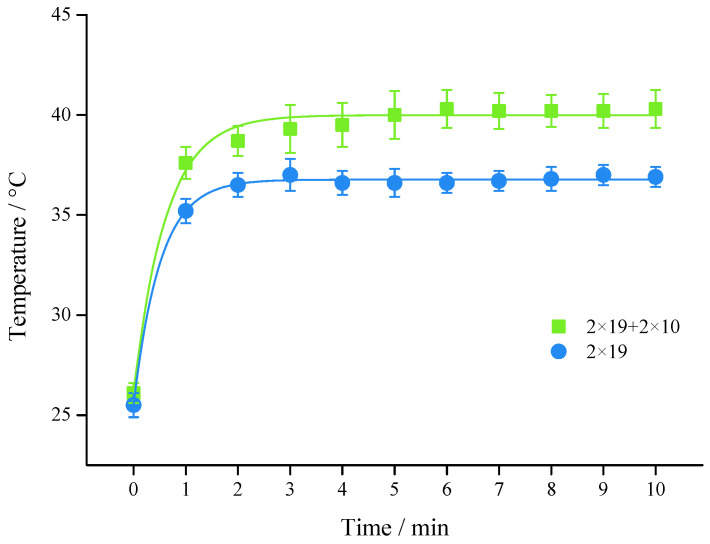
Temperature profile with different secondary coils (2 × 19 + 2 × 10 and 2 × 19) of apple juice at 800 V, 400 Hz, *d*_1_ = 1 mm, *L*_1_ = 10 cm.

The results in Figure 4 demonstrated that increasing the number of parallel secondary coils caused the temperature rise. The *T*_2_ and *T*_10_ in the high group (2 × 19 + 2 × 10) increased by 48.3% and 54.4% compared with the *T*_0_, while the temperature in the lower group (2 × 19) increased by 43.1% and 44.7%. This was due to the increase of the *N*_S_, that was equivalent to the parallel connection of multiple power sources in the link tube (Figure 2c). Because *U*_tube_ increased but *Z*_Tube_ remained unchanged, the *I*_tube_ became larger. However, more secondary coils meant higher *Z*_coil_, thus the *N_S_* could be added appropriately in the actual application.

### 3.4. Number of Parallel Link Tubes (N_Tube_)

**Figure 5:**
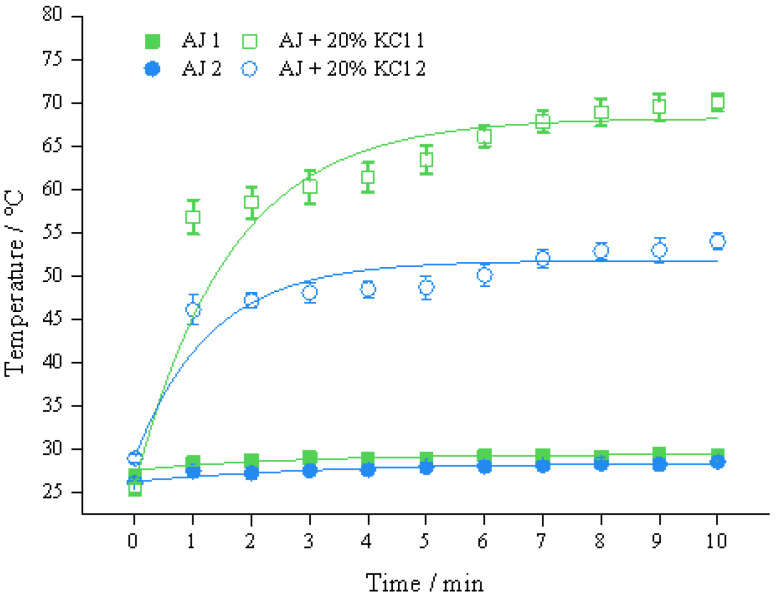
Effects of *N*_tube_ (number of parallel link tubes) on temperature profile at 800 V, 400 Hz, *d*_1_ = 2 mm, *L*_1_ = 10 cm, *N*_S_ = 2 × 19.

The temperature of the link tube decreased with the increase of *N*_Tube_, especially in the AJ + 20% KCl group (Figure 5). When *N*_Tube_ = 1, the *T*_2_ and *T*_10_ increased by 33 °C and 44.5 °C, respectively, whereas the *T*_2_ and *T*_10_ increased by 18.2 °C and 35 °C as *N*_Tube_= 2. Since the induced current was evenly distributed between the two link tubes with parallel connections, this resulted in an average distribution of the current, which reduced the heating effect. Although more link tubes would increase the capacity of the treatment, it was not suitable to adopt the parallel model because of weakening IEF.

### 3.5. The Length of the Link Tube (L_1_)

**Figure 6:**
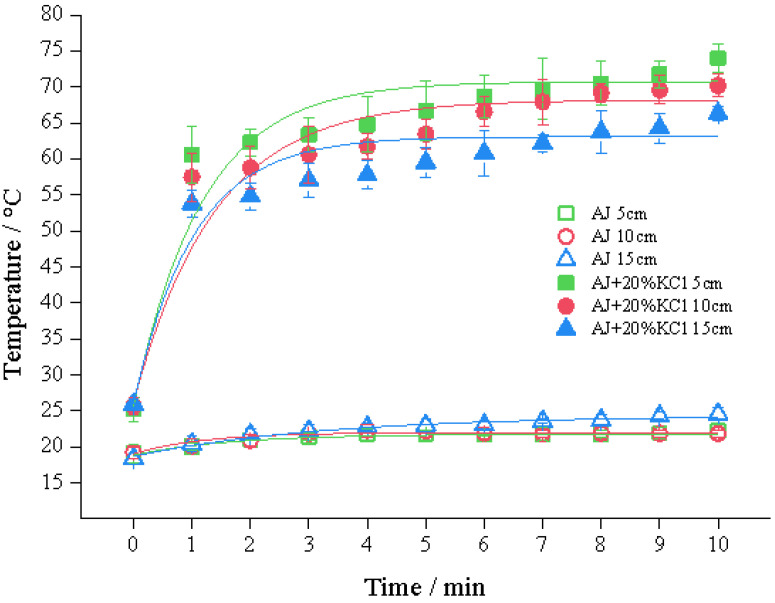
Effects of *L*_1_ on temperature at 800 V, 400 Hz, *d*_1_ = 2 mm, *N*_S_ = 2 × 19.

Data in Figure 6 show that the temperature increased with the shortened link tube filled with the AJ + 20% KCl group. The thermal effect was significantly influenced through the link tube parameters. Notably, a long tube means the prolongation of processing duration as the terminal temperature does not change. Studies on PEF and OH treatment reported that the sample could be processed with high quality [31], according to the production requirement.

### 3.6. Frequency

**Figure 7:**
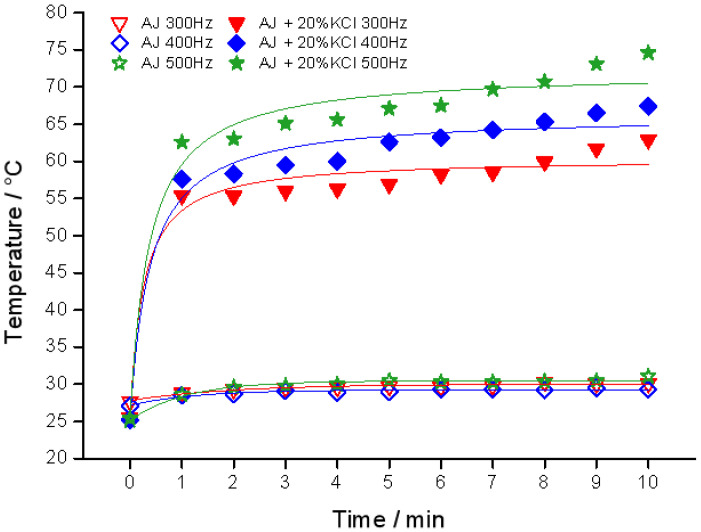
Temperature profile of different frequencies in apple juice (AJ) at 800 V, *d*_1_ = 2 mm, *L*_1_ = 10 cm, *N*_S_= 2 × 19.

The effect of the frequency on temperature is shown in Figure 7. The difference between 300 Hz, 400 Hz and 500 Hz in the AJ group was not significant in comparison to that of the AJ + 20% KCl group. The *T*_mean_ of apple juice in 300 Hz, 400 Hz and 500 Hz was 29.9 °C, 29.2 °C, and 30.4 °C, respectively, while that of the AJ + 20% KCl group was 59.2 °C, 64.2 °C and 69.8 °C, respectively. The trends in the AJ +20% KCl group indicated that the thermal effect was improved with the increase of the frequency. High frequency results in frequent molecular motion in a certain period, and the heating occurs as massive friction between molecules [32]. The thermal effect of this process are significant at higher frequency due to the motion of charged particles, which means the IEF instrument applied in the industry should be at a higher frequency.

Furthermore, the thermal effect of this inner heating could be expressed by the following equation:

(11) $$J\propto \left(a\cdot Uf\kappa_{s}+b\cdot \frac{Ns}{L_{1}d_{1}N_{\mathrm{Tube}}}\right)$$

where *a* and *b* are the scale factor of this system, *κ*_s_ is the conductivity, and *ƒ* is the frequency.

### 3.7. Physical Properties

The data in Table 3 show that the AJ sample treated at 40 kHz had higher conductivity, low impedance and resistance than the control, whereas the Brix value was not changed. Donsì et al. [33] reported that the impedance of the PEF-treated skin of Aglianico and Piedirosso grapes was reduced with the increase of the electric field at 1 kHz, but this difference gradually disappeared as the frequency increased to 10 MHz. The AJ group treated at 400 Hz had higher impedance than that of the AJ group treated at 40 kHz, which indicated that the intermediate frequency was beneficial for IEF sterilization. The low impedance means ionic species released from the cell [34], which results in the higher conductivity of the juice treated.

The pH value is related to organic compounds in apple juice [35]. The pH level of the sample treated at 40 kHz was lower than that of the AJ group, this was due to the release of organic compounds from apple cells. The study [19] pointed out IEF treatment could result in the destruction of the membrane as well as the efflux of the intracellular substance, which was also the reason why the impedance and resistance decreased.

Furthermore, the concentration of microorganisms (Table 4) revealed that the IEF system with MSCP and VDC structures could amplify the thermal effect, but the application was not enough to sterilize microorganisms in apple juice at 400 Hz. In addition, the 40 kHz and 1800 V treatment could reduce all microorganisms to undetectable levels. In order to apply the technique to the industrial production, intermediate frequency is essential.

## 4. Conclusions

This study showed that intermediate frequency and primary voltage were beneficial for the thermal effect caused by innovative induction heating, and resulted in a rapid temperature rise in the juice. The proposed structures of MSCP and VDC could significantly amplify the induced current, but the parallel connection would be adopted. In particular, the system established for the industry needs to have a smaller link tube. It was suitable for the liquid food with high conductivity. This study elucidated the sample coil that influenced the thermal effect, and enabled liquid food sterilization within one minute without a cold point. Furthermore, the IEF treatment at low frequency (300–500 Hz) had no significant effect on food properties, but the process could maintain the properties at a higher frequency (40 kHz). The study indicated that the system with MSCP and VDC structures has a potential for rapid sterilization for liquid food, through a continuous-flow and inner heating treatment. Besides, further research can be explored in terms of the nutrients of apple juice after IEF treatment at 40 kHz.

## Figures and Tables

**Table 1:** Variation of temperature with the change of *d*_1_ level at 800 V, 400 Hz, *L*_1_ = 10 cm, *N*_S_ = 2 × 19.

	*d*_1_/mm	*T*_0_/°C	*T*_2_/°C	*T*_10_/°C	*T*_mean_/°C	*V*_mean_/°C·min^−1^
AJ	1	25.5 ± 0.6 ab	36.5 ± 0.6 b	36.9 ± 0.5 b	36.7 ± 0.4 b	3.8 ± 0.3 b
	2	25.6 ± 0.5 ab	28.7 ± 0.4 c	29.3 ± 0.3 a	29.2 ± 0.1 a	1.1 ± 0.02 a
	3	25.3 ± 0.4 ab	26.6 ± 0.7 a	28.6 ± 0.5 a	28.2 ± 0.1 a	0.6 ± 0.01 a
	5	24.9 ± 0.3 a	26.0 ± 0.3 a	28.4 ± 0.3 a	29.2 ± 0.5 a	1.1 ± 0.02 a
AJ + 20% KCl	1	26.3 ± 0.3 b	–	–	–	–
	2	25.5 ± 0.2 ab	58.5 ± 1.8 d	70.0 ± 1.2 c	66.7 ± 0.3 c	11.6 ± 1.1 c
	3	26.2 ± 0.4 b	54.3 ± 0.9 e	62.9 ± 1.1 d	60.2 ± 0.9 d	9.7 ± 0.9 d
	5	25.0 ± 0.2 a	43.0 ± 0.6 f	54.0 ± 0.9 e	52.4 ± 0.7 e	7.3 ± 0.6 e

– was caused by bubbles for high temperature (over 80 °C), *p* < 0.05. Values with same letter have no significant difference (*p* < 0.05).

**Table 2:** Variation of temperature with excitation voltage at 400 Hz, *d*_1_ = 2 mm, *L*_1_ = 10 cm, *N_S_* = 2 × 19.

	*U*_P_/V	*T*_0_/°C	*T*_2_/°C	T_10_/°C	*T*_mean_/°C	*V*_mean_/°C·min^−1^
AJ	200	25.1 ± 0.4 a	26.2 ± 0.6 ab	26.7 ± 0.4 a	26.7 ± 0.1 bc	0.3 ± 0.01 a
	500	25.0 ± 0.4 a	27.1 ± 0.9 abc	28.8 ± 0.6 b	28.3 ± 0.2 d	0.8 ± 0.1 ab
	800	25.1 ± 0.3 a	28.7 ± 0.5 cd	29.3 ± 0.2 b	29.2 ± 0.3 a	1.3 ± 0.2 ab
AJ + 20% KCl	200	25.2 ± 0.5 a	29.9 ± 0.4 d	31.7 ± 0.7 c	31.1 ± 0.3 a	1.6 ± 0.1 b
	500	25.2 ± 0.4 a	37.7 ± 1.4 e	48.8 ± 1.5 d	45.7 ± 0.8 e	5.5 ± 0.3 c
	800	25.5 ± 0.3 a	58.5 ± 1.6 f	70.0 ± 1.3 e	66.7 ± 0.9 f	11.6 ± 0.5 d

*T*_0_ is the initial temperature, *T*_2_ is the temperature at 2 min, *T*_10_ is the temperature at 10 min. Values with same letter have no significant difference (*p* < 0.05).

**Table 3:** Physical properties of the samples after IEF treatment.

	pH	Brix/%	Conductivity/ms·cm^−1^	Impedance/Ω	Resistance/Ω
Control	3.87 ± 0.02 a	12.40 ± 0.12 a	2.72 ± 0.01 a	39.91 ± 1.32 a	37.47 ± 1.21 a
Treated AJ	3.84 ± 0.03 a	12.10 ± 0.20 a	2.71 ± 0.01 a	58.96 ± 1.54 b	52.87 ± 1.06 b
AJ + 20%KCl	3.56 ± 0.05 b	27.10 ± 0.26 b	224.50 ± 1.2 b	8.37 ± 0.34 c	4.77 ± 0.21 c
Treated AJ + 20%KCl	3.58 ± 0.04 b	26.70 ± 0.34 b	212.90 ± 2.3 c	8.59 ± 0.56 c	4.48 ± 0.42 c
Treated AJ 40 kHz	3.45 ± 0.01 c	12.23 ± 0.32 a	2.84 ± 0.01 d	30.42 ± 1.2 d	28.43 ± 0.8 d

Control with no IEF treatment. Treated AJ 40 kHz is the sample after 1800 V, 40 kHz treatment. Treated AJ and Treated AJ +20% KCl groups are at 800 V, 400 Hz. Values with different letters have a significant difference (*p* < 0.05).

**Table 4:** Microorganisms counting.

	*C*/CFU·mL^−1^	*N*_IEF_/%	*T*_max_*/*°C
AJ	2.40 × 10^5^	–	25.0
Treated AJ	2.38 × 10^5^	0.83	29.3
Treated AJ 40 kHz	UD	100	62.5

UD means undetected. Treated AJ 40 kHz is the sample after 1800 V, 40 kHz treatment. Treated AJ and Treated AJ +20% KCl groups are at 800 V, 400 Hz.

## Data Availability

All data presented in this study are available through the corresponding author.
